# Learning the electronic health record at the minute-scale

**DOI:** 10.64898/2026.10.04.26364562

**Published:** 2026-10-06

**Authors:** Kush Narang, Newton Addo, Jaskaran Bains, Maytal Firnberg, Sonny Tat, Anneka Hooft, Daniela Chanci Arrubla, Baraa Abed, Chandan Singh, Rishikesan Kamaleswaran, Jean Feng, Aaron Kornblith

## Abstract

Electronic health record foundation models are traditionally trained on the scale of years, days, or hours. These timescales, however, lack the minute-scale resolution required to directly guide clinical decisions at the bedside. We introduced minute-scale learning, a new paradigm for training and evaluating models, and developed MINT, a minute-scale foundation model for pediatric emergencies. MINT was pretrained and validated on 766,733 pediatric emergency department visits at five health systems, comprising 16 years of data from 10 hospitals. On minute-scale forecasting tasks, MINT outperformed and generalized to external health systems better than task-specific models (superior in 21 of 25 comparisons). MINT outperformed physicians in forecasting escalations of respiratory support. MINT demonstrated uniquely minute-scale capabilities including department-scale monitoring, dynamic risk explanations, individualized physiologic response forecasts, and hypothesis generation. Learning at the minute-scale improves performance, strengthens generalizability, and provides insights, actionability, and scientific capabilities that are not accessible at other timescales.

## Full Text Availability

The license terms selected by the author(s) for this preprint version do not permit archiving in PMC. The full text is available from the preprint server.

